# Supplementary material for: Machine learning molecular dynamics for the simulation of infrared spectra
Source: Chem Sci. 2017 Aug 10;8(10):6924–35. doi: 10.1039/c7sc02267k (PMC5636952; doi:10.1039/c7sc02267k)
Supplement: Supplementary file 1 [file SC-008-C7SC02267K-s001.pdf]

Electronic Supplementary Material (ESI) for Chemical Science.
This journal is © The Royal Society of Chemistry 2017

Supporting Information: Machine Learning Molecular Dynamics for the Simulation of Infrared Spectra

Michael Gastegger,[a] Jörg Behler,[b] and Philipp Marquetand[a*]

1 Electronic Structure Calculations

All electronic structure calculations were carried out with the ORCA program[1]. Density functional theory calculations on the BP86[2–6] (methanol and the tripeptide) and BLYP[2–4,7] (only tripeptide) level of theory were performed using the def2-SVP basis[8] set and the RI approximation with the def2-SVP/J auxilary basis set.[9,10] B2PLYP[11] computations (n-alkanes) used the def2-TZVPP basis[8] set and were accelerated using the RI-MP2 algorithm[10] in combination with the def2-TZVPP/J[12] and def2-TZVPP/C[13] auxiliary basis sets. In both cases, SCF convergence criteria were set to tight. For B2PLYP, an integration grid of size 4 was used.

2 Adaptive Sampling Scheme

2.1 Settings

The initial divergence threshold applied to E_σ in the adaptive sampling scheme was set to 5 kcal mol^{-1} and gradually tightened to 1 kcal mol^{-1} for methanol and 3 kcal mol^{-1} for n-alkanes and tripeptide. The sampling of different conformations was done with molecular dynamics trajectories using a timestep of 0.5 fs. Temperature was kept constant at 500 K through a Berendsen thermostat[14] with a coupling constant of 100 fs. Initial velocities were drawn randomly from a Maxwell–Boltzmann distribution.[15] An ensemble of two high-dimensional neural network potentials (HDNNPs) was used for all systems. The initial HDNNPs were constructed using 50 timesteps of an *ab initio* molecular dynamics (AIMD) simulation for methanol and the tripeptide. In the case of the alkanes, fragments of a structure optimized with the Merck Molecular Force Field[16] and fragmented with a cutoff radius of 4.0 Åwere found to be sufficient. After convergence of the HDNNPs for the above mentioned threshold, sampling runs were carried out for different initial conditions. If these were found to be sufficiently stable, the threshold was decreased. Neural network (NN) architectures were adapted dynamically over the course of the selection procedure.

2.2 Performance

Using methanol as a test system, we investigate the efficacy of the adaptive sampling scheme compared to an alternative approach based on random sampling. To this end, a 30 ps molecular dynamics simulation was carried out at 500 K using the General AMBER Force Field.[17] From the 60 000 configurations sampled in this way, 245 were selected at random and their energies, forces and dipole moments were recomputed at the BP86 level. Based on these reference data points, an ensemble of two HDNNPs and a dipole model were trained in the same manner as

[a] *University of Vienna, Faculty of Chemistry, Institute of Theoretical Chemistry, Währinger Str. 17, 1090 Vienna, Austria.*
[b] *Universität Göttingen, Institut für Physikalische Chemie, Theoretische Chemie, Tammannstr. 6, 37077 Göttingen, Germany.*
[*] *E-mail: philipp.marquetand@univie.ac.at; Fax: +43 1 4277 9527; Tel: +43 1 4277 52764*

described above. The resulting ML model was then used to predict the energies, forces and dipole moments along the 60 000 geometries sampled by the BP86 dynamics simulation of methanol. Figure S1 shows the distributions of the errors between the properties predicted by the ML model and the BP86 reference.

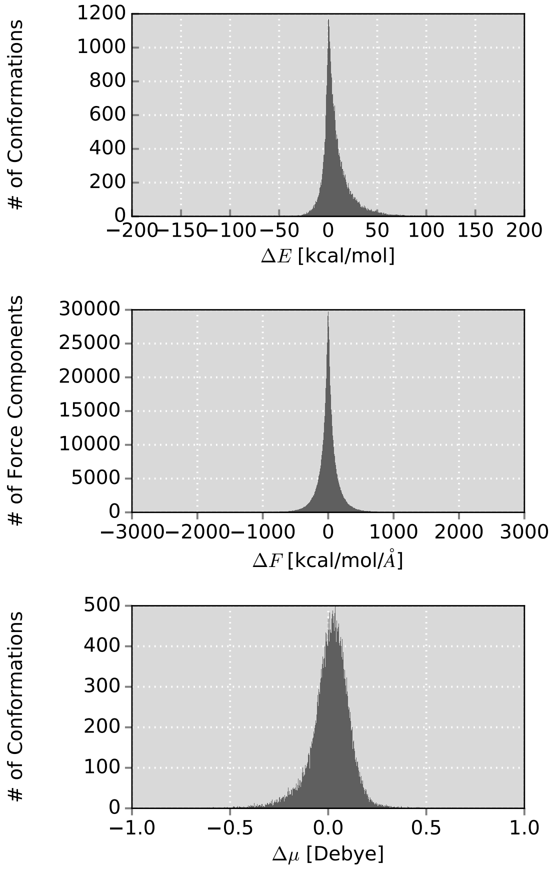

Figure S1 Distribution of errors between the BP86 reference and the predictions of the ML model based on 245 data points selected at random. The studied properties are energies (top), forces (middle) and total dipole moments (bottom). Note the increased x-axis scale compared to Fig. 4 in the main manuscript.

Compared to the ML model based on the adaptive sampling scheme, much larger deviations from the BP86 values are found for the model constructed in the above manner. While the former one exhibits MAEs for energies, forces and dipole moments of 0.048 kcal mol^{-1}, 0.533 kcal mol^{-1} Å^{-1}and 0.016 D respectively, the MAEs of the latter are 10.580 kcal mol^{-1}, 108.253 kcal mol^{-1} Å^{-1}and 0.074 D. This decrease in accuracy is mainly due to two reasons: First, the random selection of the reference data points, which can neglect rarely occurring configurations that are nevertheless important for the description of the system. Second, by using an approximate method in order to sample efficiently, the geometric properties (e.g. equilibrium bond lengths) of configurations explored in this manner can differ significantly from those that would be obtained with the reference method (which is e.g. not used directly, due to the high computational cost). As a consequence, the resulting ML potentials are only valid for e.g. bond lengths sampled via the cheap method but not necessarily relevant for the expensive electronic structure reference. Both problems are avoided in the adaptive sampling scheme, since the

HDNNPs used to sample different configurations closely resemble the reference level of theory, while the deviations between their predictions offer an uncertainty measure for the selection of new points.

The typical behavior of the adaptive sampling scheme when selecting reference configurations is shown in Figure S2 using the n-alkane as an example. While new reference data points

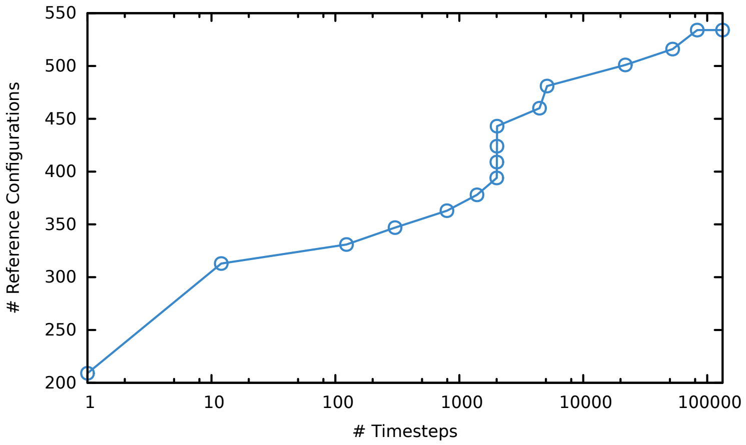

Figure S2 Cumulative number of all reference fragments (including the 209 fragments used to construct the first proto-potential) collected during the construction of the HDNNP ensemble for the n-alkanes. Fragments are sampled more frequently during the early stages or when particularly unusual situations are encountered (C-H dissociation around 2000 timesteps).

are added more frequently during the early stages of the HDNNP ensemble construction, these events become increasingly rare as the ensemble approaches convergence. An interesting effect can be observed in the vicinity of 2000 timesteps, where a particularly undersampled region of the PES is encountered (dissociation of a hydrogen atom) and the sampling frequency increases again for a short time.

2.3 HDNNP ensembles

Table S1 shows the performance of ML models for methanol employing ensembles of different sizes. The respective MAEs for energies, forces and dipole moments (once again computed along the BP86 trajectory) are averaged for all possible ensembles of size N constructed from the best 5 HDNNPs and dipole models. In the case of the energies and forces, improvements in accuracy

Table S1 Average MAEs of energies, forces and dipole moments along the BP86 trajectory obtained by ML ensembles based on all possible combinations using N of the best five HDNNPs and dipole models. Reference data points were selected using the adaptive sampling scheme.

# HDNNPs	MAE E [kcal mol^{-1}]	MAE F [kcal mol^{-1} Å^{-1}]	MAE μ [D]
1	0.056	0.597	0.0170
2	0.051	0.544	0.0169
3	0.049	0.525	0.0169
4	0.048	0.515	0.0169
5	0.047	0.509	0.0168

can be gained by increasing the number of HDNNPs and dipole NNs. This gain is largest for the step from $N = 1$ to $N = 2$ but decreases quickly when more models are added. The dipole moment model does not seem to profit significantly from using more than one predictor.

As can be seen for $N = 1$, the basic building components of the above models already exhibit excellent accuracy and consequently the improvements introduced by larger ensembles are relatively small. However, in the case of more approximate models (e.g. during the early stages of the adaptive sampling scheme), ensembles offer a much greater advantage. This effect is demonstrated by performing the above analysis for ML models based on the randomly selected reference data points (see Table S2). Here, the MAEs of the predicted energies and forces are

Table S2 Average MAEs of energies, forces and dipole moments along the BP86 trajectory obtained by ML ensembles based on all possible combinations using N of the best five HDNNPs and dipole models. Reference data points were selected using the random sampling scheme described above.

# HDNNPs	MAE E [kcal mol^{-1}]	MAE F [kcal mol^{-1} Å^{-1}]	MAE μ [D]
1	18.097	167.537	0.0958
2	12.843	119.395	0.0872
3	10.628	98.439	0.0849
4	9.307	85.957	0.0840
5	8.372	77.085	0.0835

reduced to less than half of their initial value when changing the number of base predictors from $N = 1$ to $N = 5$, which is close to the expected reduction of $\frac{1}{\sqrt{5}} \approx 0.44$. However, only minor improvements are once again observed in the case of the dipole moments.

Based on the above observations, we chose two HDNNPs to model the potential energies of all systems, as this offers the best trade off between an improved accuracy and the associated increase in simulation time. Since the dipole moment models do not seem to profit from using more predictors and are only used once a converged reference data set has been obtained, no ensembles are employed in this case.

3 Infrared Spectra

IR spectra were obtained with molecular dynamics simulations employing the same timestep and initialization as for the sampling procedure (see Section 2). After an initial equilibration period (3ps for methanol, 5ps otherwise), simulations were run at 300 K in case of methanol and n-alkanes (for 30 and 50 ps) and 350 K for the tripeptide (for 50ps). Temperature was kept constant using a massive Nóse-Hoover-chain thermostat[18–20] during equilibration and a standard Nóse-Hoover thermostat[18,20] during production runs. In both cases a chain length of 3 and a relaxation time of 100 fs were used. Forces and energies were obtained using a HDNNP ensemble of size two. In addition to HDNNP accelerated dynamics, AIMD simulations were carried out for methanol using the BP86 level of theory described above. Dipole-dipole autocorrelation functions were computed according to the Wiener-Khinchin theorem[21] for an autocorrelation depth of 2048 fs. A combination of a Hann window function[22] and zero-padding were applied to the autocorrelation functions before Fourier transformation in order to improve

the quality of the final IR spectra.

4 High Dimensional Neural Network Potentials (HDNNPs)

Training and construction of the HDNNPs was carried out with the RuNNer program. HDNNPs were trained for 100 epochs with the element decoupled Kalman filter, using a forgetting schedule with $\lambda_0 = 0.95$ and $\lambda_k = 0.995$.[23] The hidden layers of the elemental NNs employed the softplus activation function:

$$\sigma_p(x) = \log\left(e^x + 1\right), \tag{1}$$

while the final layer applied a linear transformation. Weights were initialized using the procedure described in Reference[24]. Molecular forces were included in the optimization procedure with a weighting factor of $\eta = 10$. To accelerate training, all ACSF were scaled to a range between -1 and 1 and the energies of the free atoms were subtracted from the target potential energies. In addition, an adaptive filter threshold of 0.9 times the current RMSE was employed to energy and force updates. The final models were determined using cross validation combined with an early stopping schedule, with 10 percent of the data in the validation set.[25]

The neural network architectures employed in the final HDNNPs are given in Table S3. These networks were selected automatically during the adaptive sampling runs from a pool of ten different architectures, based on their performance in reproducing reference energies and forces.

Table S3 Architectures of the elemental neural networks used in the final composite ML models. Given are the number of nodes in every hidden layer. The input dimensions are the lengths of the ACSF input vectors (see Table S6), output dimension is one in all cases.

System	HDNN 1	HDNN 2	DIPOLE
Methanol	30-30	20-20	100-100
n-Alkanes	35-35	20-20	50-50
Tripeptide	20-10-10-10-5	25-15-5	50-50

For a detailed discussion of the atom-centered symmetry functions used to describe the chemical environments in the HDNNPs and the dipole model, see Section 7.

5 Dipole Moment Model

The NN dipole models were implemented in python[26] using the `numpy`[27] and `theano`[28] packages. The chemical environments of the individual atoms were described with the same ACSFs as for the HDNNPs (see Section 7). Weights were once again initialized using the above procedure. Unlike in the HDNNPs, hyperbolic tangent nonlinearities were used in the hidden layers. For all systems, training was carried out for 10 000 epochs using the ADAM optimizer[29] with parameter settings of $\varepsilon = 10^{-8}$, $\beta_1 = 0.9$ and $\beta_2 = 0.999$. Individual learning rates and L_2 regularization strengths are given in Table S4.

Once again, the final models were determined using cross validation combined with an early stopping schedule (10 percent of data as validation data). The final NN architectures can be found in Table S3.

Table S4 Learning rates α and strength of the L_2 regularization used for training the dipole models.

System	α	L_2
Methanol	0.0000100	0.0001
n-Alkanes	0.0000429	0.0001
Tripeptide	0.0001389	0.0001

6 Static Vibrational Frequencies and IR intensities

In order to gain further insights into the accuracy of the developed ML models, the static IR spectra predicted by these models were compared to the corresponding electronic structure spectra. To this end, finite difference computations were used to obtain the Hessians and dipole moment derivatives necessary to calculate the normal mode frequencies and IR intensities. Prior to these computations, all molecular structures were optimized using the respective electronic structure methods and ML models. The deviations between the static ML and electronic structure spectra with respect to vibrational frequencies and absorption intensities can be found in Table S5. In the case of the $C_{69}H_{140}$, no static electronic structure spectra could be obtained due to the large number and prohibitive computational cost of the individual finite difference calculations.

Table S5 Comparison of the static vibrational frequencies and IR intensities obtained with finite difference electronic structure computations and the respective ML models. In addition to the MAEs, the deviation of each property is also given as a percentage of the overall range of the observed values. Column three shows the number of displacements required to compute the static electronic structure spectra if molecular forces are utilized, while column four contains the number of configurations used to construct the corresponding ML models. The ratio between these two quantities is given in column five.

System	# Atoms	# Displ.	# Samples	Ratio	MAE			
					$\tilde{\omega}$ [cm^{-1}]	$\tilde{\omega}$ [%]	I [km mol^{-1}]	I [%]
Methanol	6	36	245	6.81	9.76	0.26	8.09	6.74
Butane	14	84	534	6.36	18.18	0.58	8.38	6.59
$C_{69}H_{140}$	209	1254		0.43	-	-	-	-
Ala_3^+-NH_2	34	204			13.96	0.39	53.68	3.17
Ala_3^+-NH_3	34	204	717	1.17	13.12	0.36	41.08	7.01
Ala_3^+-FOL	34	204			18.41	0.52	46.48	3.83

As can be seen, all ML models exhibit an excellent agreement with their electronic structure reference. Especially the normal mode frequencies are reproduced to a high degree of accuracy, exhibiting a maximum MAE of 18.41 cm^{-1} (0.053 kcal mol^{-1}). Slightly larger, but still comparatively small errors are observed for the IR intensities. The main reason for this effect is the way static IR intensities are computed. These intensities are proportional to square of the derivatives of the dipole moments with respect to the normal mode coordinates of the different vibrational modes. In order to obtain these derivatives, the Cartesian derivatives need to be transformed using the unitary matrix which diagonalizes the Hessian. As a consequence, the static IR inten-

sities are not only susceptible to fluctuations in the dipole moment model, but also deviations in the HDNNPs used to predict the Hessian. This accumulation of error leads to the slightly increased MAEs observed for the IR intensities.

In addition to a comparison of the static spectra, Table S5 also gives the ratio between the reference samples used to construct the different ML models and the number of electronic structure finite difference computations. As can be seen, with a growing number of atoms contained in a system and/or the configurations considered for a spectrum, the ratio of the overall required electronic structure calculations begins to shift in favor of our ML model. Especially impressive is the case of $C_{69}H_{140}$, where the ML model needs less than half the points required for a finite difference Hessian. However, even the larger ratios obtained for butane and methanol should be taken with a grain of salt: While the finite difference spectrum only provides information on the regions of the PES very close to the minimum configuration, the information contained in the ML model is far more extensive. As a consequence, the ML model can be used for molecular dynamics simulations and is hence able to account for temperature effects and vibrational anharmonicities, which are completely neglected in the finite difference spectra. In order to provide an accurate perspective on the relative computational efficiency of the ML model and the performance of the sampling scheme, the number of reference points would have to be compared to the number of configurations sampled by respective AIMD simulation of an IR spectrum. In the case of methanol, the ML model is based on 245 reference points, while the AIMD trajectory encompasses 66 000 steps including equilibration. The resulting ratio is 0.0037, providing an excellent perspective on the significant advantage in computational efficiency provided by the present ML model.

7 Atom-Centered Symmetry Functions

The local chemical environment of the different atoms in methanol and the n-alkanes is characterized via radial symmetry functions of the type

$$G_i^{\mathrm{rad}} = \sum_{j \neq i}^{N_{\mathrm{atoms}}} e^{-\eta (R_{ij} - R_{\mathrm{s}})^2} f_{\mathrm{c}}(R_{ij}), \tag{2}$$

and angular symmetry functions

$$G_i^{\mathrm{ang}} = 2^{1-\zeta} \sum_{j,k \neq i}^{N_{\mathrm{atoms}}} \big(1 + \lambda \cos(\theta_{ijk})\big)\, e^{-\eta (R_{ij}^2 + R_{ik}^2 + R_{jk}^2)} \times f_{\mathrm{c}}(R_{ij}) f_{\mathrm{c}}(R_{ik}) f_{\mathrm{c}}(R_{jk}). \tag{3}$$

R_{ij} is the distance between atoms i and j (analogous also for atoms k), R_s is the offset of the Gaussian function. η, ζ and λ are parameters which determine the overall shape of the symmetry functions. f_c is a cutoff function introduced to limit the description of the local environment to the chemically relevant regions and is defined as

$$f_{\mathrm{c}}(R_{ij}) = \begin{cases} \frac{1}{2}\left[\cos\left(\frac{\pi R_{ij}}{R_{\mathrm{c}}}\right) + 1\right], & R_{ij} \leq R_{\mathrm{c}} \\ 0, & R_{ij} > R_{\mathrm{c}}, \end{cases}$$

with R_c as the cutoff radius. For a more detailed discussion of the different symmetry functions, see Reference[30]. In the present work, cutoff radii of 6.35 Å, 4.00 Å and 5.00 Å were used for methanol, the n-alkanes and the protonated tripeptide respectively.

In case of the protonated alanine tripeptide, a slightly modified version of Equation 3 was used to describe the angular atomic environment:

$$G_i^{\mathrm{ang2}} = 2^{1-\zeta} \sum_{j,k\neq i}^{N_{\mathrm{atoms}}} \left(1+\lambda\cos(\theta_{ijk})\right) e^{-\eta(R_{ij}^2+R_{ik}^2)} \times f_{\mathrm{c}}(R_{ij}) f_{\mathrm{c}}(R_{ik}). \tag{4}$$

The overall composition of the symmetry functions for the different molecular systems can be found in Table S6. The individual parameters of the radial and angular symmetry functions used to describe the methanol molecule are given in Tables S7 to S12, those for the n-alkanes can be found in Tables S13 to S16 and the parameters for the tripeptide functions are given in Tables S17 to S24. HDNNPs and dipole models use exactly the same symmetry functions.

Table S6 Number of symmetry functions used to describe the atomic chemical environments in the different molecules.

System	Element	# Radial	# Angular	# Total
Methanol	H	3	16	19
	C	2	8	10
	O	2	8	10
n-Alkanes	H	8	24	32
	C	8	24	32
Tripeptide	H	16	40	56
	C	16	40	56
	O	16	40	56
	N	16	40	56

References

[1] F. Neese, *WIREs Comput. Mol. Sci.*, 2012, **2**, 73–78.

[2] A. D. Becke, *Phys. Rev. A*, 1988, **38**, 3098–3100.

[3] P. A. M. Dirac, *Proc. R. Soc. A*, 1929, **123**, 714–733.

[4] J. P. Perdew, *Phys. Rev. B*, 1986, **33**, 8822–8824.

[5] S. H. Vosko, L. Wilk and M. Nusair, *Can. J. Phys.*, 1980, **58**, 1200–1211.

[6] J. C. Slater, *Phys. Rev.*, 1951, **81**, 385–390.

[7] C. Lee, W. Yang and R. G. Parr, *Phys. Rev. B*, 1988, **37**, 785–789.

[8] F. Weigend and R. Ahlrichs, *Phys. Chem. Chem. Phys.*, 2005, **7**, 3297–3305.

[9] K. Eichkorn, O. Treutler, H. Öhm, M. Häser and R. Ahlrichs, *Chem. Phys. Lett.*, 1995, **240**, 283–290.

[10] O. Vahtras, J. Almlöf and M. W. Feyereisen, *Chem. Phys. Lett.*, 1993, **213**, 514–518.

[11] S. Grimme, *J. Chem. Phys.*, 2006, **124**, 034108.

[12] F. Weigend, *Phys. Chem. Chem. Phys.*, 2006, **8**, 1057–1065.

[13] F. Weigend, M. Häser, H. Patzelt and R. Ahlrichs, *Chem. Phys. Lett.*, 1998, **294**, 143 – 152.

[14] H. J. C. Berendsen, J. P. M. Postma, W. F. van Gunsteren, A. DiNola and J. R. Haak, *J. Chem. Phys.*, 1984, **81**, 3684–3690.

[15] R. C. Tolman, *The Principles of Statistical Mechanics*, Dover Publications, New York, New edn, 2010.

[16] T. A. Halgren, *J. Comput. Chem.*, 1996, **17**, 490–519.

[17] J. Wang, R. M. Wolf, J. W. Caldwell, P. A. Kollman and D. A. Case, *J. Comput. Chem.*, 2004, **25**, 1157–1174.

[18] W. G. Hoover, *Phys. Rev. A*, 1985, **31**, 1695–1697.

[19] Martyna, Glenn J., M. L. Klein and M. Tuckerman, *J. Chem. Phys.*, 1992, **97**, 2635–2643.

[20] S. Nosé, *Mol. Phys.*, 1984, **52**, 255–268.

[21] N. Wiener, *Acta Math.*, 1930, **55**, 117–258.

[22] R. B. Blackman and J. W. Tukey, *Bell Syst. Tech. J.*, 1958, **37**, 185–282.

[23] M. Gastegger and P. Marquetand, *J. Chem. Theory Comput.*, 2015, **11**, 2187–2198.

[24] X. Glorot and Y. Bengio, Proceedings of the Thirteenth International Conference on Artificial Intelligence and Statistics, Chia Laguna Resort, Sardinia, Italy, 2010, pp. 249–256.

Table S7 Parameters of the radial symmetry functions describing the chemical environment of H atoms in methanol.

No.	Neighbor	η [Bohr^{-2}]	R_s [Bohr]	R_c [Bohr]
1	C	0.01000000	0.0	12.00000
2	H	0.01000000	0.0	12.00000
3	O	0.01000000	0.0	12.00000

Table S8 Parameters of the angular symmetry functions describing the chemical environment of H atoms in methanol.

No.	Neighbors	η [Bohr^{-2}]	λ	ζ	R_c [Bohr]
4	C H	0.00800000	-1.0	1.0	12.00000
5	C H	0.00800000	-1.0	4.0	12.00000
6	C H	0.00800000	1.0	1.0	12.00000
7	C H	0.00800000	1.0	4.0	12.00000
8	C O	0.00800000	-1.0	1.0	12.00000
9	C O	0.00800000	-1.0	4.0	12.00000
10	C O	0.00800000	1.0	1.0	12.00000
11	C O	0.00800000	1.0	4.0	12.00000
12	H H	0.00800000	-1.0	1.0	12.00000
13	H H	0.00800000	-1.0	4.0	12.00000
14	H H	0.00800000	1.0	1.0	12.00000
15	H H	0.00800000	1.0	4.0	12.00000
16	O H	0.00800000	-1.0	1.0	12.00000
17	O H	0.00800000	-1.0	4.0	12.00000
18	O H	0.00800000	1.0	1.0	12.00000
19	O H	0.00800000	1.0	4.0	12.00000

[25] C. M. Bishop, *Pattern Recognition and Machine Learning*, Springer, New York, 1st edn, 2006.

[26] G. van Rossum and F. L. Drake (eds), *Python Reference Manual*, PythonLabs, Virginia, USA, 2001; Available at `http://www.python.org` (accessed date 06.04.2017).

[27] S. van der Walt, S. C. Colbert and G. Varoquaux, *Comput. Sci. Eng.*, 2011, **13**, 22–30.

[28] J. Bergstra, O. Breuleux, F. Bastien, P. Lamblin, R. Pascanu, G. Desjardins, J. Turian, D. Warde Farley and Y. Bengio, Proceedings of the Python for Scientific Computing Conference (SciPy), 2010.

[29] D. P. Kingma and J. Ba, *CoRR*, 2014, **abs/1412.6980**, 0000.

[30] J. Behler, *J. Chem. Phys.*, 2011, **134**, 074106.

Table S9 Parameters of the radial symmetry functions describing the chemical environment of C atoms in methanol.

No.	Neighbor	η [Bohr^{-2}]	R_s [Bohr]	R_c [Bohr]
1	H	0.01000000	0.0	12.00000
2	O	0.01000000	0.0	12.00000

Table S10 Parameters of the angular symmetry functions describing the chemical environment of C atoms in methanol.

No.	Neighbors	η [Bohr^{-2}]	λ	ζ	R_c [Bohr]
3	H H	0.00800000	-1.0	1.0	12.00000
4	H H	0.00800000	-1.0	4.0	12.00000
5	H H	0.00800000	1.0	1.0	12.00000
6	H H	0.00800000	1.0	4.0	12.00000
7	O H	0.00800000	-1.0	1.0	12.00000
8	O H	0.00800000	-1.0	4.0	12.00000
9	O H	0.00800000	1.0	1.0	12.00000
10	O H	0.00800000	1.0	4.0	12.00000

Table S11 Parameters of the radial symmetry functions describing the chemical environment of O atoms in methanol.

No.	Neighbor	η [Bohr^{-2}]	R_s [Bohr]	R_c [Bohr]
1	C	0.01000000	0.0	12.00000
2	H	0.01000000	0.0	12.00000

Table S12 Parameters of the angular symmetry functions describing the chemical environment of O atoms in methanol.

No.	Neighbors	η [Bohr^{-2}]	λ	ζ	R_c [Bohr]
3	C H	0.00800000	-1.0	1.0	12.00000
4	C H	0.00800000	-1.0	4.0	12.00000
5	C H	0.00800000	1.0	1.0	12.00000
6	C H	0.00800000	1.0	4.0	12.00000
7	H H	0.00800000	-1.0	1.0	12.00000
8	H H	0.00800000	-1.0	4.0	12.00000
9	H H	0.00800000	1.0	1.0	12.00000
10	H H	0.00800000	1.0	4.0	12.00000

Table S13 Parameters of the radial symmetry functions describing the chemical environment of H atoms in the n-alkanes.

No.	Neighbor	η [Bohr^{-2}]	R_s [Bohr]	R_c [Bohr]
1	C	0.12700000	0.0	7.55890
2	C	0.06350000	0.0	7.55890
3	C	0.03175000	0.0	7.55890
4	C	0.01587000	0.0	7.55890
5	H	0.04469000	0.0	7.55890
6	H	0.02235000	0.0	7.55890
7	H	0.01117290	0.0	7.55890
8	H	0.00558645	0.0	7.55890

Table S14 Parameters of the angular symmetry functions describing the chemical environment of H atoms in the n-alkanes.

No.	Neighbors	η [Bohr^{-2}]	λ	ζ	R_c [Bohr]
9	C C	0.05981000	-1.0	1.0	7.55890
10	C C	0.02991000	-1.0	1.0	7.55890
11	C C	0.01495304	-1.0	1.0	7.55890
12	C C	0.00074765	-1.0	4.0	7.55890
13	C C	0.05981000	1.0	1.0	7.55890
14	C C	0.02991000	1.0	1.0	7.55890
15	C C	0.01495304	1.0	1.0	7.55890
16	C C	0.00074765	1.0	4.0	7.55890
17	C H	0.12700000	-1.0	1.0	7.55890
18	C H	0.06350000	-1.0	1.0	7.55890
19	C H	0.03175000	-1.0	1.0	7.55890
20	C H	0.01587000	-1.0	4.0	7.55890
21	C H	0.12700000	1.0	1.0	7.55890
22	C H	0.06350000	1.0	1.0	7.55890
23	C H	0.03175000	1.0	1.0	7.55890
24	C H	0.01587000	1.0	4.0	7.55890
25	H H	0.04469000	-1.0	1.0	7.55890
26	H H	0.02235000	-1.0	1.0	7.55890
27	H H	0.01117290	-1.0	1.0	7.55890
28	H H	0.00558645	-1.0	4.0	7.55890
29	H H	0.04469000	1.0	1.0	7.55890
30	H H	0.02235000	1.0	1.0	7.55890
31	H H	0.01117290	1.0	1.0	7.55890
32	H H	0.00558645	1.0	4.0	7.55890

Table S15 Parameters of the radial symmetry functions describing the chemical environment of C atoms in the n-alkanes.

No.	Neighbor	η [Bohr^{-2}]	R_s [Bohr]	R_c [Bohr]
1	C	0.05981000	0.0	7.55890
2	C	0.02991000	0.0	7.55890
3	C	0.01495304	0.0	7.55890
4	C	0.00074765	0.0	7.55890
5	H	0.12700000	0.0	7.55890
6	H	0.06350000	0.0	7.55890
7	H	0.03175000	0.0	7.55890
8	H	0.01587000	0.0	7.55890

Table S16 Parameters of the angular symmetry functions describing the chemical environment of C atoms in the n-alkanes.

No.	Neighbors	η [Bohr^{-2}]	λ	ζ	R_c [Bohr]
9	C C	0.05981000	-1.0	1.0	7.55890
10	C C	0.02991000	-1.0	1.0	7.55890
11	C C	0.01495304	-1.0	1.0	7.55890
12	C C	0.00074765	-1.0	4.0	7.55890
13	C C	0.05981000	1.0	1.0	7.55890
14	C C	0.02991000	1.0	1.0	7.55890
15	C C	0.01495304	1.0	1.0	7.55890
16	C C	0.00074765	1.0	4.0	7.55890
17	C H	0.12700000	-1.0	1.0	7.55890
18	C H	0.06350000	-1.0	1.0	7.55890
19	C H	0.03175000	-1.0	1.0	7.55890
20	C H	0.01587000	-1.0	4.0	7.55890
21	C H	0.12700000	1.0	1.0	7.55890
22	C H	0.06350000	1.0	1.0	7.55890
23	C H	0.03175000	1.0	1.0	7.55890
24	C H	0.01587000	1.0	4.0	7.55890
25	H H	0.04469000	-1.0	1.0	7.55890
26	H H	0.02235000	-1.0	1.0	7.55890
27	H H	0.01117290	-1.0	1.0	7.55890
28	H H	0.00558645	-1.0	4.0	7.55890
29	H H	0.04469000	1.0	1.0	7.55890
30	H H	0.02235000	1.0	1.0	7.55890
31	H H	0.01117290	1.0	1.0	7.55890
32	H H	0.00558645	1.0	4.0	7.55890

Table S17 Parameters of the radial symmetry functions describing the chemical environment of H atoms in the protonated alanine tripeptide.

No.	Neighbor	η [Bohr^{-2}]	R_s [Bohr]	R_c [Bohr]
1	C	0.29648286	0.0	9.44863
2	C	0.19471693	0.0	9.44863
3	C	0.11166594	0.0	9.44863
4	C	0.02642717	0.0	9.44863
5	H	0.24717164	0.0	9.44863
6	H	0.18772949	0.0	9.44863
7	H	0.03772191	0.0	9.44863
8	H	0.01605820	0.0	9.44863
9	N	0.22678375	0.0	9.44863
10	N	0.09913557	0.0	9.44863
11	N	0.02385194	0.0	9.44863
12	N	0.01784315	0.0	9.44863
13	O	0.41844859	0.0	9.44863
14	O	0.11188355	0.0	9.44863
15	O	0.02715895	0.0	9.44863
16	O	0.00385156	0.0	9.44863

Table S18 Parameters of the angular symmetry functions describing the chemical environment of H atoms in the protonated alanine tripeptide.

No.	Neighbors	η [Bohr^{-2}]	λ	ζ	R_c [Bohr]
17	C C	0.02179822	-1.0	1.0	9.44863
18	C C	0.00300501	-1.0	1.0	9.44863
19	C C	0.20112380	1.0	1.0	9.44863
20	C C	0.00510347	1.0	1.0	9.44863
21	C N	0.07025182	-1.0	1.0	9.44863
22	C N	0.03569861	-1.0	1.0	9.44863
23	C N	0.12329650	1.0	1.0	9.44863
24	C N	0.02679261	1.0	1.0	9.44863
25	C O	0.04793390	-1.0	1.0	9.44863
26	C O	0.01378368	-1.0	1.0	9.44863
27	C O	0.38427813	1.0	1.0	9.44863
28	C O	0.05345156	1.0	1.0	9.44863
29	H C	0.04626174	-1.0	1.0	9.44863
30	H C	0.04250331	-1.0	1.0	9.44863
31	H C	0.24563666	1.0	1.0	9.44863
32	H C	0.05945747	1.0	1.0	9.44863
33	H H	0.17698146	-1.0	1.0	9.44863
34	H H	0.00184611	-1.0	1.0	9.44863
35	H H	0.01432978	1.0	1.0	9.44863
36	H H	0.01049936	1.0	1.0	9.44863
37	H N	0.04468575	-1.0	1.0	9.44863
38	H N	0.00251361	-1.0	1.0	9.44863
39	H N	0.13619324	1.0	1.0	9.44863
40	H N	0.00744905	1.0	1.0	9.44863
41	H O	0.18318457	-1.0	1.0	9.44863
42	H O	0.00538124	-1.0	1.0	9.44863
43	H O	0.05206716	1.0	1.0	9.44863
44	H O	0.01580180	1.0	1.0	9.44863
45	N N	0.09475159	-1.0	1.0	9.44863
46	N N	0.00386485	-1.0	1.0	9.44863
47	N N	0.04567493	1.0	1.0	9.44863
48	N N	0.01883538	1.0	1.0	9.44863
49	N O	0.08358792	-1.0	1.0	9.44863
50	N O	0.01239754	-1.0	1.0	9.44863
51	N O	0.15744426	1.0	1.0	9.44863
52	N O	0.02570670	1.0	1.0	9.44863
53	O O	0.05405209	-1.0	1.0	9.44863
54	O O	0.02726588	-1.0	1.0	9.44863
55	O O	0.06052873	1.0	1.0	9.44863
56	O O	0.03554242	1.0	1.0	9.44863

Table S19 Parameters of the radial symmetry functions describing the chemical environment of C atoms in the protonated alanine tripeptide.

No.	Neighbor	η [Bohr^{-2}]	R_s [Bohr]	R_c [Bohr]
1	C	0.28163727	0.0	9.44863
2	C	0.08662835	0.0	9.44863
3	C	0.06987626	0.0	9.44863
4	C	0.04638289	0.0	9.44863
5	H	0.15076950	0.0	9.44863
6	H	0.11133857	0.0	9.44863
7	H	0.04236020	0.0	9.44863
8	H	0.00993211	0.0	9.44863
9	N	0.23394702	0.0	9.44863
10	N	0.22710726	0.0	9.44863
11	N	0.10334032	0.0	9.44863
12	N	0.08037534	0.0	9.44863
13	O	0.11582560	0.0	9.44863
14	O	0.04129150	0.0	9.44863
15	O	0.02701535	0.0	9.44863
16	O	0.00724349	0.0	9.44863

Table S20 Parameters of the angular symmetry functions describing the chemical environment of C atoms in the protonated alanine tripeptide.

No.	Neighbors	η [Bohr^{-2}]	λ	ζ	R_c [Bohr]
17	C C	0.11805618	-1.0	1.0	9.44863
18	C C	0.02967452	-1.0	1.0	9.44863
19	C C	0.12674502	1.0	1.0	9.44863
20	C C	0.05395903	1.0	1.0	9.44863
21	C N	0.10461706	-1.0	1.0	9.44863
22	C N	0.08186352	-1.0	1.0	9.44863
23	C N	0.09828284	1.0	1.0	9.44863
24	C N	0.06216240	1.0	1.0	9.44863
25	C O	0.09804122	-1.0	1.0	9.44863
26	C O	0.01251918	-1.0	1.0	9.44863
27	C O	0.13475312	1.0	1.0	9.44863
28	C O	0.01934041	1.0	1.0	9.44863
29	H C	0.05557461	-1.0	1.0	9.44863
30	H C	0.04149527	-1.0	1.0	9.44863
31	H C	0.18441234	1.0	1.0	9.44863
32	H C	0.05221292	1.0	1.0	9.44863
33	H H	0.11841617	-1.0	1.0	9.44863
34	H H	0.03604772	-1.0	1.0	9.44863
35	H H	0.03826696	1.0	1.0	9.44863
36	H H	0.03823943	1.0	1.0	9.44863
37	H N	0.05127798	-1.0	1.0	9.44863
38	H N	0.00673929	-1.0	1.0	9.44863
39	H N	0.25414291	1.0	1.0	9.44863
40	H N	0.11119231	1.0	1.0	9.44863
41	H O	0.04638956	-1.0	1.0	9.44863
42	H O	0.03851434	-1.0	1.0	9.44863
43	H O	0.19372937	1.0	1.0	9.44863
44	H O	0.00461013	1.0	1.0	9.44863
45	N N	0.12631207	-1.0	1.0	9.44863
46	N N	0.03748320	-1.0	1.0	9.44863
47	N N	0.02260529	1.0	1.0	9.44863
48	N N	0.01237789	1.0	1.0	9.44863
49	N O	0.04102916	-1.0	1.0	9.44863
50	N O	0.00409384	-1.0	1.0	9.44863
51	N O	0.07834422	1.0	1.0	9.44863
52	N O	0.03834974	1.0	1.0	9.44863
53	O O	0.24335588	-1.0	1.0	9.44863
54	O O	0.03634173	-1.0	1.0	9.44863
55	O O	0.07709959	1.0	1.0	9.44863
56	O O	0.04604883	1.0	1.0	9.44863

Table S21 Parameters of the radial symmetry functions describing the chemical environment of O atoms in the protonated alanine tripeptide.

No.	Neighbor	η [Bohr^{-2}]	R_s [Bohr]	R_c [Bohr]
1	C	0.32191107	0.0	9.44863
2	C	0.08762364	0.0	9.44863
3	C	0.06723966	0.0	9.44863
4	C	0.03408388	0.0	9.44863
5	H	0.05205290	0.0	9.44863
6	H	0.02127948	0.0	9.44863
7	H	0.01891170	0.0	9.44863
8	H	0.00221202	0.0	9.44863
9	N	0.07402704	0.0	9.44863
10	N	0.06896284	0.0	9.44863
11	N	0.04599393	0.0	9.44863
12	N	0.01382052	0.0	9.44863
13	O	0.08513965	0.0	9.44863
14	O	0.05539260	0.0	9.44863
15	O	0.00966241	0.0	9.44863
16	O	0.00656202	0.0	9.44863

Table S22 Parameters of the angular symmetry functions describing the chemical environment of O atoms in the protonated alanine tripeptide.

No.	Neighbors	η [Bohr^{-2}]	λ	ζ	R_c [Bohr]
17	C C	0.07069046	-1.0	1.0	9.44863
18	C C	0.02859012	-1.0	1.0	9.44863
19	C C	0.18721891	1.0	1.0	9.44863
20	C C	0.02611385	1.0	1.0	9.44863
21	C N	0.04303934	-1.0	1.0	9.44863
22	C N	0.00748429	-1.0	1.0	9.44863
23	C N	0.21557530	1.0	1.0	9.44863
24	C N	0.03305416	1.0	1.0	9.44863
25	C O	0.13357540	-1.0	1.0	9.44863
26	C O	0.01626814	-1.0	1.0	9.44863
27	C O	0.03300501	1.0	1.0	9.44863
28	C O	0.01299665	1.0	1.0	9.44863
29	H C	0.43705856	-1.0	1.0	9.44863
30	H C	0.00653748	-1.0	1.0	9.44863
31	H C	0.07583808	1.0	1.0	9.44863
32	H C	0.07264994	1.0	1.0	9.44863
33	H H	0.08344736	-1.0	1.0	9.44863
34	H H	0.00772657	-1.0	1.0	9.44863
35	H H	0.05603848	1.0	1.0	9.44863
36	H H	0.05176458	1.0	1.0	9.44863
37	H N	0.05393470	-1.0	1.0	9.44863
38	H N	0.01001503	-1.0	1.0	9.44863
39	H N	0.08256963	1.0	1.0	9.44863
40	H N	0.07321780	1.0	1.0	9.44863
41	H O	0.03335767	-1.0	1.0	9.44863
42	H O	0.00718154	-1.0	1.0	9.44863
43	H O	0.00408989	1.0	1.0	9.44863
44	H O	0.00068442	1.0	1.0	9.44863
45	N N	0.04559482	-1.0	1.0	9.44863
46	N N	0.01567034	-1.0	1.0	9.44863
47	N N	0.32011232	1.0	1.0	9.44863
48	N N	0.00926972	1.0	1.0	9.44863
49	N O	0.00095330	-1.0	1.0	9.44863
50	N O	0.00093466	-1.0	1.0	9.44863
51	N O	0.01260151	1.0	1.0	9.44863
52	N O	0.00258750	1.0	1.0	9.44863
53	O O	0.05900071	-1.0	1.0	9.44863
54	O O	0.00733462	-1.0	1.0	9.44863
55	O O	0.08023656	1.0	1.0	9.44863
56	O O	0.03402109	1.0	1.0	9.44863

Table S23 Parameters of the radial symmetry functions describing the chemical environment of N atoms in the protonated alanine tripeptide.

No.	Neighbor	η [Bohr^{-2}]	R_s [Bohr]	R_c [Bohr]
1	C	0.18475708	0.0	9.44863
2	C	0.16257344	0.0	9.44863
3	C	0.02203683	0.0	9.44863
4	C	0.00238280	0.0	9.44863
5	H	0.34084482	0.0	9.44863
6	H	0.19274488	0.0	9.44863
7	H	0.13980154	0.0	9.44863
8	H	0.00026158	0.0	9.44863
9	N	0.27083832	0.0	9.44863
10	N	0.05266323	0.0	9.44863
11	N	0.01368073	0.0	9.44863
12	N	0.00864736	0.0	9.44863
13	O	0.07724499	0.0	9.44863
14	O	0.03262546	0.0	9.44863
15	O	0.02067713	0.0	9.44863
16	O	0.01541025	0.0	9.44863

Table S24 Parameters of the angular symmetry functions describing the chemical environment of N atoms in the protonated alanine tripeptide.

No.	Neighbors	η [Bohr^{-2}]	λ	ζ	R_c [Bohr]
17	C C	0.14432377	-1.0	1.0	9.44863
18	C C	0.08530500	-1.0	1.0	9.44863
19	C C	0.15256024	1.0	1.0	9.44863
20	C C	0.03791440	1.0	1.0	9.44863
21	C N	0.05116226	-1.0	1.0	9.44863
22	C N	0.01427118	-1.0	1.0	9.44863
23	C N	0.08357299	1.0	1.0	9.44863
24	C N	0.02367891	1.0	1.0	9.44863
25	C O	0.02920770	-1.0	1.0	9.44863
26	C O	0.01295525	-1.0	1.0	9.44863
27	C O	0.04535736	1.0	1.0	9.44863
28	C O	0.01642360	1.0	1.0	9.44863
29	H C	0.03953409	-1.0	1.0	9.44863
30	H C	0.03217121	-1.0	1.0	9.44863
31	H C	0.13144784	1.0	1.0	9.44863
32	H C	0.01468040	1.0	1.0	9.44863
33	H H	0.04671755	-1.0	1.0	9.44863
34	H H	0.00726313	-1.0	1.0	9.44863
35	H H	0.06397314	1.0	1.0	9.44863
36	H H	0.03527693	1.0	1.0	9.44863
37	H N	0.04937304	-1.0	1.0	9.44863
38	H N	0.02888120	-1.0	1.0	9.44863
39	H N	0.04639814	1.0	1.0	9.44863
40	H N	0.03283510	1.0	1.0	9.44863
41	H O	0.11842255	-1.0	1.0	9.44863
42	H O	0.07205809	-1.0	1.0	9.44863
43	H O	0.04835207	1.0	1.0	9.44863
44	H O	0.00730130	1.0	1.0	9.44863
45	N N	0.05464277	-1.0	1.0	9.44863
46	N N	0.00561318	-1.0	1.0	9.44863
47	N N	0.01982970	1.0	1.0	9.44863
48	N N	0.01189888	1.0	1.0	9.44863
49	N O	0.08219679	-1.0	1.0	9.44863
50	N O	0.03324282	-1.0	1.0	9.44863
51	N O	0.07943351	1.0	1.0	9.44863
52	N O	0.03876848	1.0	1.0	9.44863
53	O O	0.03098453	-1.0	1.0	9.44863
54	O O	0.01899243	-1.0	1.0	9.44863
55	O O	0.14583125	1.0	1.0	9.44863
56	O O	0.00018801	1.0	1.0	9.44863
